# Pregnancy and estrogen enhance neural progenitor-cell proliferation in the vomeronasal sensory epithelium

**DOI:** 10.1186/s12915-015-0211-8

**Published:** 2015-11-30

**Authors:** Livio Oboti, Ximena Ibarra-Soria, Anabel Pérez-Gómez, Andreas Schmid, Martina Pyrski, Nicole Paschek, Sarah Kircher, Darren W. Logan, Trese Leinders-Zufall, Frank Zufall, Pablo Chamero

**Affiliations:** Department of Physiology, and Center for Integrative Physiology and Molecular Medicine, University of Saarland School of Medicine, 66421 Homburg, Germany; Wellcome Trust Sanger Institute, Wellcome Trust Genome Campus, Hinxton, Cambridge CB10 1SA UK; Monell Chemical Senses Center, 3500 Market Street, Philadelphia, Pennsylvania 19104 USA; Present address: Center for Neuroscience Research, Children’s National Health System, 20010 Washington, DC, USA; Present address: Laboratoire de Physiologie de la Reproduction & des Comportments, UMR 7247 INRA-CNRS-Université François Rabelais, F-37380 Nouzilly, France

**Keywords:** Adult neurogenesis, Esr1, Estrogen, Hormone receptors, Pregnancy, Vomeronasal organ, Vomeronasal receptors

## Abstract

**Background:**

The hormonal state during the estrus cycle or pregnancy produces alterations on female olfactory perception that are accompanied by specific maternal behaviors, but it is unclear how sex hormones act on the olfactory system to enable these sensory changes.

**Results:**

Herein, we show that the production of neuronal progenitors is stimulated in the vomeronasal organ (VNO) epithelium of female mice during a late phase of pregnancy. Using a wide range of molecular markers that cover the whole VNO cell maturation process in combination with Ca^2+^ imaging in early postmitotic neurons, we show that newly generated VNO cells adopt morphological and functional properties of mature sensory neurons. A fraction of these newly generated cells project their axons to the olfactory forebrain, extend dendrites that contact the VNO lumen, and can detect peptides and urinary proteins shown to contain pheromone activity. High-throughput RNA-sequencing reveals concomitant differences in gene expression in the VNO transcriptomes of pregnant females. These include relative increases in expression of 20 vomeronasal receptors, of which 17 belong to the V1R subfamily, and may therefore be considered as candidate receptors for mediating maternal behaviors. We identify the expression of several hormone receptors in the VNO of which estrogen receptor α (Esr1) is directly localized to neural progenitors. Administration of sustained high levels of estrogen, but not progesterone, is sufficient to stimulate vomeronasal progenitor cell proliferation in the VNO epithelium.

**Conclusions:**

Peripheral olfactory neurogenesis driven by estrogen may contribute to modulate sensory perception and adaptive VNO-dependent behaviors during pregnancy and early motherhood.

**Electronic supplementary material:**

The online version of this article (doi:10.1186/s12915-015-0211-8) contains supplementary material, which is available to authorized users.

## Background

The vertebrate olfactory system is characterized by a distinctive plastic capacity of cell renewal and axon rewiring that continues during adulthood. This represents an exception within the central nervous system where neurogenesis is largely restricted to embryonic development and early postnatal stages. Adult neurogenesis occurs almost exclusively in three specific areas, the hippocampal subgranular zone, the subventricular zone (SVZ) and the olfactory epithelia [1–5]. Two of these regions, the SVZ and olfactory epithelia, supply new neurons directly to parts of the olfactory system in form of olfactory bulb interneurons and olfactory sensory neurons, respectively [4–6]. Within peripheral olfactory epithelia, the vomeronasal organ (VNO) conveys chemosensory information, such as pheromones and predator odors, that drive social and reproductive behaviors [7–9]. Vomeronasal sensory neurons (VSNs) are constantly renewed throughout the life of the animal [10] by neurons generated from stem cells located at the lateral and basal margins of the mature sensory epithelium [11–14]. Most of these margin newborn cells differentiate into mature VSNs rarely undergoing apoptosis [15], and project their axons to the accessory olfactory bulb (AOB) [16]. Whether these newly generated cells become functional VSNs and thus are capable of transducing chemosensory cues has not been determined.

In the olfactory epithelia, adult neurogenesis is often seen as a static, merely restorative process, not regarded as a real mechanism for plasticity. However, every aspect of adult-born cell production is carefully regulated and modulated, strongly suggesting that the olfactory system can tailor its production of new neurons to match the demands of its environment. Newborn neurons in the VNO derive from neural progenitors expressing *Mash1* and *Ngn1* genes [17, 18]. During their proliferation phase, the majority of these progenitors express Ki-67 (antigen identified by monoclonal antibody Ki-67) and proliferating cell nuclear antigen (PCNA) proteins associated with the cell cycle [19]. Postmitotic, immature VSNs express markers associated with cytoskeletal remodeling such as doublecortin (*Dcx*), β-tubulin III, and GAP43, NCAM/OCAM adhesion molecules, and other lineage-related genes differentially expressed during the maturation process, including *Bcl11b/Ctip2*, *Gnao1*, and *Gnai2* [13, 17, 20]. *Dcx* is widely expressed in non-mitotic immature VSNs and their axons during the whole VSN maturation period [21]. Immature neurons migrate towards more superficial and central layers of the VNO epithelium and mature into bipolar neurons characterized by the expression of olfactory marker protein (OMP) and vomeronasal receptors (VR) of the V1R and V2R families [17, 22, 23]. Proliferation of VSN precursors can be activated by a number of intrinsic signals, such as growth factors [21, 24], but also by external stimuli including exposure to urinary compounds [20]. However, it is not known whether adult neurogenesis in the VNO operates in response to specific physiological conditions to modulate VNO-dependent sensory function and behavior.

Adult neurogenesis in the brain has been shown to be regulated by a number of physiological, pathological, and environmental factors, including stress, synaptic activity, hormonal status, injury, and odor exposure [25]. Hormonal changes that occur during pregnancy modulate forebrain and olfactory neurogenesis. Prolactin, luteinizing hormone, and estradiol have been shown to promote neurogenesis and cell survival in the brain [26–33]. This is important for the display of maternal behavior [28, 29, 34], because olfactory discrimination and memory, both facilitated by adult-born neurons in the brain [35], are critical for offspring recognition and care [36]. With few exceptions [37], hormonal and pregnancy effects on VNO neurogenesis have not been investigated, yet several lines of evidence associate sex hormones with modulatory effects on sensory function. Hormones and hormone derivatives activate subsets of VSNs [38–40] and regulate VSN sensory perception in female mice [41]. Modulatory effects of sensory perception by motherhood and lactation have been recently reported in other systems. For example, hormonal changes associated with motherhood and pup care seem to enable plastic changes in sensory perception of the auditory system [42, 43].

Therefore, the aim of the present study was to elucidate the mechanisms controlling adult neurogenesis in the mouse VNO. First, we performed a molecular characterization of the maturation phase of newly generated cells in the vomeronasal sensory epithelium. Second, we evaluated the functional sensory properties of these cells. Third, we found that neurogenesis in the VNO is enhanced at the end of pregnancy. Fourth, we determined that neuroblasts in the VNO express estrogen receptor α (*Esr1*) and that sustained high levels of the hormone estrogen enable a higher proliferation rate. These results show that neurogenesis in the VNO epithelium can be modulated by an ovarian hormone, offering important insight to understand pregnancy-evoked changes in olfactory perception and behavior.

## Results

### Adult neurogenesis in the vomeronasal organ

**Fig. 1.**
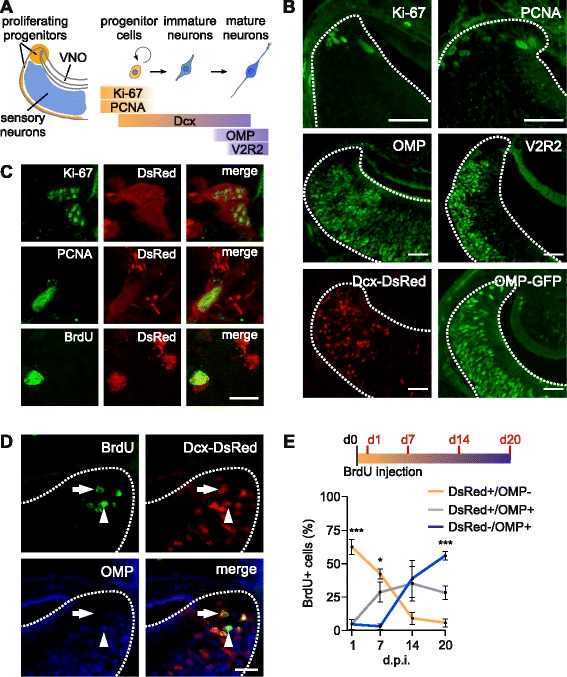
Neurogenesis and development of vomeronasal sensory neurons (VSNs). (**a**) Schematic of VSN maturation and markers used. Ki-67/proliferating cell nuclear antigen (PCNA)-positive proliferating progenitors in the marginal zones of the vomeronasal organ (VNO; orange) develop to immature doublecortin (Dcx)-expressing neurons and mature VSNs in the central VNO (blue) expressing olfactory marker protein (OMP) and vomeronasal receptors (VRs). Ki-67 is a nuclear antigen expressed during the whole cell cycle except at G_0_. PCNA participates in DNA synthesis. Dcx is a microtubule-associated protein expressed in immature neurons. OMP is expressed in mature olfactory and vomeronasal sensory neurons. V2R2 antibody recognizes family-C V2Rs expressed in most mature basal VNO neurons. (**b**) Coronal VNO tissue slices labeled with antibodies against the early proliferation markers Ki-67 and PCNA (top panels) reveal expression on the VNO marginal zones, whereas in maturity, the markers OMP and V2R2 (center) localize on the center of the sensory epithelium on all or basal VSNs, respectively. Bottom panels: endogenous expression on OMP-GFP and Dcx-DsRed transgenic mice in the VNO. Dotted lines show the limits of the sensory epithelium. Scale bars, 50 μm. (**c**) Double-labeling of Ki-67, PCNA and BrdU (1 day post-injection) immunostainings (green) with Dcx-DsRed in individual cells of the VNO epithelial margins. Scale bar, 10 μm. (**d**) Triple-labeling with antibodies recognizing BrdU (green) and OMP (blue) with the DsRed (Red) on VNO coronal slices of Dcx-DsRed mice 14 days after injection with BrdU. Newborn VSNs can be identified in an early maturity stage by double labeling of BrdU and DsRed (arrow) and in a later stage by triple labeling of BrdU/DsRed/OMP (arrowhead). Dotted lines show the limits of the sensory epithelium. Scale bar, 25 μm. (**e**) Fate-mapping analysis of BrdU^+^ cells in the VNO epithelium quantified at 1, 7, 14 and 21 days post injection (d.p.i.) in VNO coronal slices of Dcx-DsRed mice immunolabelled with BrdU and OMP, as shown in (**d**). OMP^+^ cells increase, while DsRed^+^ and OMP^–^ cells decrease over time. About 50 % of the newly generated cells reach a mature phenotype in 20 days. One-way ANOVA: F_2,8_ = 2.15–56.3; ***P <0.005 (1 d.p.i.; DsRed^+^/OMP^–^ vs. DsRed^+^/OMP^+^ and DsRed^–^/OMP^+^), *P <0.05 (7 d.p.i.; DsRed^–^/OMP^+^ vs. DsRed^+^/OMP^+^ and DsRed^+^/OMP^–^), ***P <0.005 (20 d.p.i.; DsRed^–^/OMP^+^ vs. DsRed^+^/OMP^–^ and DsRed^+^/OMP^+^), n = 3 mice per time point

To investigate the cellular basis of adult neurogenesis in the mouse vomeronasal epithelium, we used a range of molecular markers (Fig. 1a) expressed at different stages of neuron generation and maturation: Ki-67 and PCNA, which are nuclear proteins present during DNA synthesis in actively proliferating cells [19]; *Dcx*, which is expressed by neuronal precursor cells and immature neurons for 2–3 weeks after cell division [44]; OMP, which is expressed in all mature sensory neurons, and type-2 vomeronasal receptor (V2R2), present in mature neurons of the basal layer of the VNO [22, 45, 46]; bromodeoxyuridine, 5-bromo-2’-deoxyuridine (BrdU), a synthetic analog of thymidine that is incorporated into replicating DNA, was used to label cells that were actively proliferating at the time of administration [19]. To visualize these markers in the VNO epithelium we used either immunolocalization (Ki-67, PCNA, BrdU, OMP, and V2R2; Fig. 1b, top and central panels) or mouse strains expressing a fluorescent reporter under the control of a marker protein: Dcx-DsRed [47] and OMP-GFP mice [48] (Fig. 1b, bottom panels). Imaging on adult VNO tissue slices revealed that progenitor cells expressing Ki-67, PCNA, or BrdU (1 day post injection) are restricted to the marginal zones of the epithelium (Fig. 1a, b, d) whereas OMP-expressing mature neurons localize in both marginal and central zones (Fig. 1a, b). This is consistent with previous studies identifying these marginal VNO regions as active neurogenic sites (Fig. 1a) [11, 13–15, 17]. Immature neurons expressing Dcx-DsRed are predominantly distributed marginally although occupying a larger zone than Ki-67/PCNA-labeled cells (Fig. 1b), indicative of active migration to more central areas during cell maturation [49]. Higher magnification analysis at single cell-resolution revealed that fractions of Ki-67, PCNA, and BrdU positive cells co-express Dcx-DsRed (Fig. 1c). However, cells positive for these early proliferation markers do not co-localize with V2R2 and OMP^+^ cells (data not shown). Hence, Dcx-DsRed is widely expressed through neuron maturation from early proliferative stages to newly differentiated mature VSNs.

Next, we analyzed the time course of VSN generation and maturation in adult Dcx-DsRed female mice. We injected mice with 100 mg/kg BrdU and analyzed its incorporation in the VNO of four animal groups: 1, 7, 14, and 20 days post-injection (Fig. 1e). In addition to Dcx-DsRed reporter expression, VNO sections were immunolabelled for BrdU (green) and OMP (blue; Fig. 1d). BrdU^+^ cells were classified in three developmental stages: (1) postmitotic progenitor cells that are positive for BrdU and DsRed, but negative for OMP; (2) newly differentiated neurons positive for BrdU, DsRed, and OMP; and (3) mature neurons positive for BrdU and OMP, but negative for DsRed (Fig. 1e). Cells positive for BrdU and negative for DsRed and OMP probably account for either actively dividing progenitors or early differentiating glial cells. Cell quantification revealed that at day 1 after BrdU injection the most abundant BrdU immunoreactivity (62 %) corresponds to postmitotic progenitors (*P* <0.005), indicating a rapid proliferation cycle. These cells decreased sharply after 7–14 days, and by day 20 represented only ~5 % of the total BrdU^+^ cells. Newly differentiated neurons (positive for both DsRed and OMP) were nearly absent during the first day, but sharply increased to 28–35 % at 7–14 days, and were maintained stable (28 %) at 20 days. By contrast, fully mature neurons (OMP^+^, DsRed^–^) were absent during the first 7 days, rapidly growing to 38 % after 14 days, and becoming the most abundant cell type (55 %) at 20 days (*P* <0.005, n = 12; Fig. 1e). Thus, most VSNs maturate within a period of 14–20 days and only a very small fraction of progenitor cells remain undifferentiated during this time period.

### Newly born vomeronasal cells give rise to functional sensory neurons

**Fig. 2.**
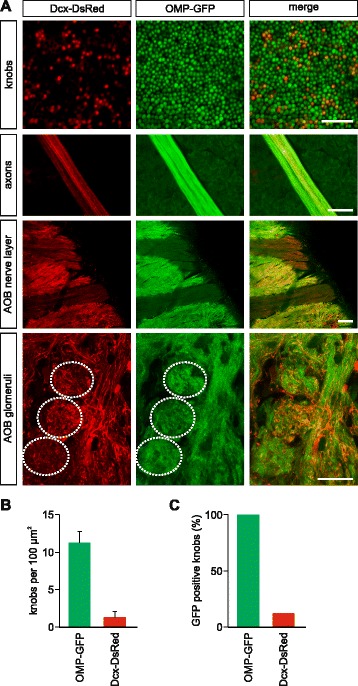
Proliferating progenitors in the vomeronasal organ (VNO) exhibit sensory neuron morphology. (**a**) Imaging of knob layer on the luminal surface of the VNO sensory epithelium of a doublecortin (Dcx)-DsRed/OMP-GFP double transgenic mouse (top panels). DsRed^+^ knobs intermingle within the more mature population of GFP^+^ vomeronasal sensory neurons (VSNs). Scale bar, 20 μm. DsRed^+^ axonal processes (second panels) are detected and extend towards the accessory olfactory bulb (AOB) together with mature GFP^+^ fibers. Scale bar, 50 μm. Medial view of the caudal part of the olfactory bulb (third panels) where the axons from the VNO bend off laterally towards the AOB. GFP expression in VSNs is markedly lower than in the main olfactory epithelium and therefore axon bundles from the VNO appear as substantially darker. In DsRed images, VSN axon bundles are easily identified by their conspicuous parallel alignment. Scale bar, 100 μm. Sagittal view of AOB glomeruli (bottom panel). DsRed^+^ axons originating in the nerve layer terminate in several glomeruli (dotted circles), visualized by OMP-GFP fluorescence. Scale bar, 20 μm. (**b**) Density measurements of DsRed^+^ and GFP^+^ knobs reveal a sparser density of immature knobs compared to the mature population. (**c**) Quantification of randomly sampled knobs shows that double-labeled GFP/DsRed^+^ knobs represent 12.14 % of the total GFP^+^ knob population. Values in (**b**) and (**c**) were calculated using 12 areas from six Dxc-DsRed/OMP-GFP mice; mean ± standard deviation (SD) = 1.41 ± 0.72 DsRed and 11.25 ± 1.55 OMP-GFP knobs per 100 μm^2^. DsRed-GFP/GFP ratio = 12.14 %, 2,649 double positive knobs

We next asked whether newly generated cells become functional sensory neurons in the vomeronasal neuroepithelium. First, we studied the general morphology and axon conveyance of Dcx-DsRed^+^ cells. To provide a direct and simultaneous comparison of Dcx and OMP labeling, we crossed Dcx-DsRed mice with OMP-GFP mice (Dxc-DsRed/OMP-GFP; Fig. 2). Utilizing *en face* confocal imaging in a VNO whole-mount preparation [50], we visualized VSN knobs at the dendritic tips of Dxc-DsRed/OMP-GFP mice (Fig. 2a). The *en face* view of the vomeronasal epithelium reveals a homogeneous and punctate distribution of OMP-GFP-expressing VSNs (Fig. 2a). Notably, Dcx-DsRed^+^ knobs were also present at dendritic tips of a substantial number of VSNs (Fig. 2a). OMP-GFP^+^ knobs were 5–10-fold more abundant than Dcx-DsRed^+^ knobs (Fig. 2b; GFP knob density: 11.25 knobs/100 μm^2^; DsRed knob density: 1.41 knobs/100 μm^2^; n = 6) and the level of overlap between OMP-GFP and Dcx-DsRed represented ~12 % of GFP^+^ knobs (Fig. 2c; DsRed-GFP/GFP ratio = 12.14 %, 2,649 double positive knobs). Furthermore, imaging of VNO axons and the AOB nerve layer illustrates strong enrichment of Dcx-DsRed axons in both structures (Fig. 2a). Dcx-DsRed^+^ axons deriving from the nerve layer terminate into AOB glomeruli that were identified by co-localization with OMP-GFP (Fig. 2a, bottom). Virtually all glomeruli in the anterior and posterior AOB show Dcx-DsRed^+^ fibers. These data show that Dcx-DsRed^+^ VSNs can extend their dendrites into the VNO lumen and send axonal projections that reach the AOB glomeruli.

**Fig. 3.**
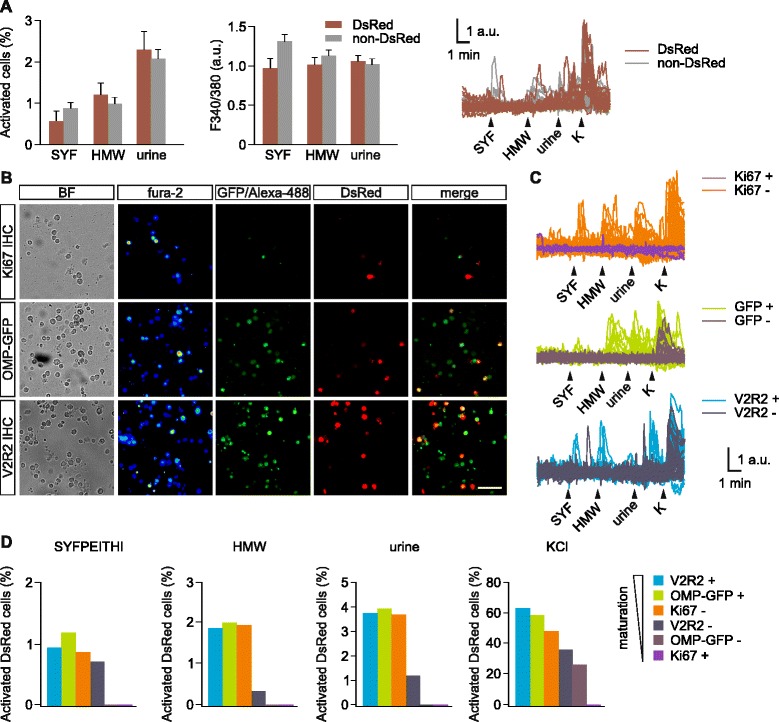
Vomeronasal sensory neurons (VSN) activation by pheromonal compounds at different maturity stages. (**a**) Dissociated vomeronasal organ (VNO) cells from doublecortin (Dcx)-DsRed mice, either DsRed^+^ (red bars) and DsRed^–^ (gray bars), show intracellular calcium transients in response to the major histocompatibility complex-derived peptide SYFPEITHI (SYF, 10^−11^ M), whole male urine (1:300 dilution) and its high molecular weight fraction (1:300). Both the number of activated cells (two-way ANOVA: F_1,149_ = 0.04; *P* = 0.85) and the intracellular fura-2 fluorescent increases (two-way ANOVA: F_1,186_ = 1.3; *P* = 0.26) indicate no differences in the response rate of DsRed positive vs. negative cell populations for all three stimuli. Example traces of Ca^2+^ transients in single cells in response to the stimuli. High KCl (90 mM) solution was delivered at the end of the experiment to assess cell viability. (**b**) Post-hoc immunolabeling for Ki-67, V2R2 or endogenous OMP-GFP in Ca^2+^-imaged VNO cells. Representative images of VNO cells taken with transmission light, bright field, fura-2 pseudocolor fluorescence 340/380 nm ratio (fura-2), 488 nm excitation light of green-labeled secondary antibodies, or OMP-GFP fluorescence (GFP/Alexa-488), DsRed fluorescence (DsRed), and merged ex 488 nm/DsRed color images (merged). OMP-GFP/Dcx-DsRed double transgenic mice were used to label OMP-GFP cells. Scale bar, 50 μm. (**c**) Representative fura-2 ratio traces of DsRed^+^ cells labeled with Ki-67, OMP-GFP, or V2R2. Responses of cells positive vs. negative for Ki-67, OMP-GFP, and V2R2 are shown. (**d**) Percentage of activated DsRed cells classified by marker expression. Near-maximum response rates to the three stimuli are reached in cells positive for V2R2 and OMP and negative for Ki-67. No responses were found in cells positive for Ki-67. Cells negative for OMP-GFP showed responses only to high K^+^

Since maturating VSNs have access to chemostimuli in the VNO lumen and can deliver the olfactory information to the AOB, we next asked whether these cells are functional sensory neurons capable of detecting chemosignals. We analyzed stimulus-induced activity in the VNO of Dcx-DsRed mice using ratiometric Ca^2+^ imaging on freshly dissociated VSNs [51, 52]. We compared the response pattern of Dcx-DsRed-positive vs. -negative cells in the same VSN preparation in response to three stimuli: (1) the major histocompatibility complex-peptide SYFPEITHI (10^−11^ M); (2) fresh adult male C57Bl/6 urine (diluted 1:300); and (3) its high molecular weight fraction (HMW; 1:300), containing major urinary proteins [51, 52]. These stimuli have been previously established as VSNs stimuli [51, 53]. Activation by these three stimuli was detected in ~1–3 % of the 6,186 cells screened, taken from 25 mice. Importantly, all three stimuli induced similar levels of cell activation in both Dcx-DsRed^+^ and Dcx-DsRed^–^ cells (*P* = 0.85; Fig. 3a), suggesting that Dcx-DsRed^+^ cells exhibit functional sensory properties.

To assess a more precise maturity status of Dcx-DsRed + cells, we examined the expression of OMP, V2R2, and Ki-67 using post-hoc immunostaining of responsive VSNs directly in the Ca^2+^ imaging recording chamber [51, 52] in independent parallel experiments. To determine the maturation stage, we classified the cells based on the combination of the specific markers at the time: cells positive for either OMP or V2R2 or negative for Ki-67 reached a late maturation stage, whereas cells positive for Ki-67 or negative for OMP and V2R2 were in an early stage (Fig. 3b). We imaged 2,173 (Ki-67), 2,715 (OMP-GFP) and 2,233 (V2R2) cells taken from 10, 7, and 7 mice in three parallel experiments and calculated the number of Dcx-DsRed^+^ cells activated by the three stimuli. We observed near-maximum rates of activation for SYFPEITHI, HMW, and whole urine in cells labeled with late maturity markers V2R2 and OMP and negative for Ki-67 (Fig. 3b, c). Immature cells that were either negative for OMP or positive for Ki-67 showed no responses to any of the three pheromone stimuli. Ki-67^+^ cells are a minority (~2 %) within the total pool of Dcx-DsRed^+^, and therefore this lack or reduced responsiveness remains unnoticed when averaging all Dcx-DsRed^+^ responses (Fig. 3a). Cells negative for V2R2 showed a 2- to 5-fold reduction in cell activity (Fig. 3c, d), suggesting intermediate maturation. Stimulation with depolarizing high K^+^ (90 mM) solution confirmed the presence of voltage-dependent Ca^2+^ channels in these cells (Fig. 3c, d). All cell groups exhibited activation to high K^+^ (27 % or higher) except for Ki-67^+^ cells (0 %), confirming that neuron-like features are not yet fully developed in these cells. Together, these results indicate that newly generated Dcx-DsRed^+^ VSNs become chemosensitive neurons and that this process occurs at a late maturation stage subsequent to the expression of OMP and V2R2, but not in immature Ki-67^+^ cells.

### VNO neurogenesis is enhanced during pregnancy

**Fig. 4.**
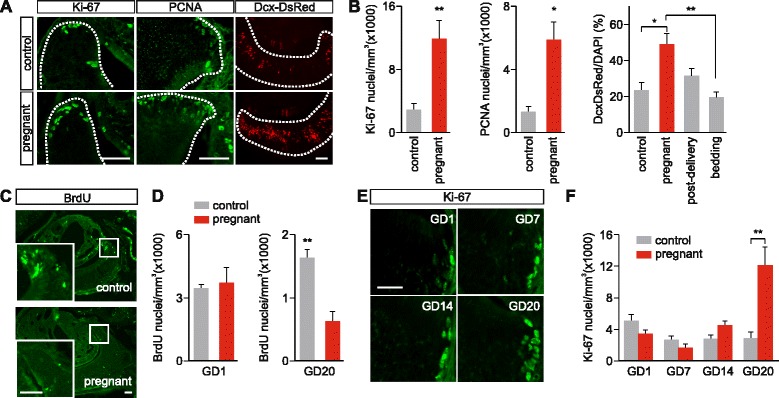
Pregnancy enhances vomeronasal organ (VNO) neurogenesis. (**a**) Coronal VNO sections immunolabelled with Ki-67, proliferating cell nuclear antigen (PCNA), and endogenous doublecortin (Dcx)-DsRed in 19/20-day pregnant females and control females of same age. Dotted lines show the limits of the VNO sensory epithelium. Scale bars, 50 μm. (**b**) Quantification of the densities of Ki-67- and PCNA-labeled cells, and relative abundance of Dcx-DsRed^+^ cells vs. total cells labeled with the nuclear dye DAPI; 19/20-day pregnancy induced increase in immature neurons labeled with all markers. Ki-67, PCNA, Student’s *t*-test, *P <0.05; **P <0.01. Dcx-DsRed, ANOVA: F_3,27_ = 4.44; *P <0.05, **P <0.01. The increase of Dcx-DsRed cells is not induced by exposure to male bedding for 20 days (bedding vs. control, *P* = 0.873) and fades 3 days after delivery (post-delivery vs. control, *P* = 0.77). (**c**) Immunostaining of BrdU^+^ cells in the VNOs of control and GD20 pregnant mice injected with BrdU at GD0. Scale bars, 50 μm. (**d**) Quantification of BrdU^+^ cells in the VNOs of GD1 and GD20 pregnant mice and their non-pregnant controls injected with BrdU at GD0. The overall density of newborn cells marked with BrdU decreases at GD20, but not at GD1. Student’s *t*-test, **P <0.01. (**e**) VNO coronal sections labeled with Ki-67 antibody at different gestation days (GD). Scale bar, 20 μm. (**f**) Quantification of Ki-67^+^ nuclei density in the VNO at the different gestation days. Two-way ANOVA: F_1,33_ = 5.38; **P <0.01; n = 34

In mammals, adult stem cell division is physiologically stimulated during pregnancy in the forebrain SVZ and haematopoietic tissues which seem to favor the display of postpartum and maternal behaviors [28, 29, 31, 54]. To test whether VNO neurogenesis is upregulated during pregnancy, we examined the abundance of Ki-67, PCNA, and Dcx-DsRed^+^ cells in the VNOs of pregnant females at gestation days (GDs) 19–20 and non-pregnant controls of the same age (Fig. 4a). We found that pregnant mice had significantly more cells positive for the three markers in the VNO after 19–20 days of gestation (Fig. 4b and Additional file 1: ratio of DsRed^+^ cells pregnant/control 1.75 ± 0.26; *P <0.05; **P <0.01, n = 3 pregnant and 13 control mice; PCNA: control 1,256 ± 361, n = 3; pregnant 5,838 ± 1,090, n = 3 *P <0.05; Ki-67, control, 2,860 ± 734, n = 6; pregnant, 12,111 ± 2,680, n = 4 **P <0.01), indicating a pregnancy-induced increase in neurogenesis. The increase on Dcx-DsRed^+^ cells occurred in the whole VNO neuroepithelium with no obvious apical/basal gradients. Female mice used in these experiments were previously exposed to adult males in order to be inseminated (see Methods). To verify that contact to male odors does not influence VNO stem cell proliferation, we exposed control-naïve females to male bedding for 20 days and counted the number of Dcx-DsRed cells (Fig. 4b). Bedding-exposed females showed control levels of Dcx-DsRed cells in the VNO (*P* = 0.873; n = 9 mice) indicating that male-derived odors did not increase the generation of new VSNs. We further determined whether high rates of immature neurons stay stable over time or decrease after parturition. We quantified the number of Dcx-DsRed^+^ cells 3 days after delivery (Fig. 4b) and observed no significant differences compared to controls (*P* = 0.77; n = 3 mice), indicating that the number of Dcx-DsRed^+^ returns to pre-pregnancy levels after parturition.

The increased neurogenesis could result from higher proliferation or, alternatively, from a higher cell survival rate. To distinguish between these possibilities, we injected females with BrdU at GD0 and examined immunoreactivity at GD20 (Fig. 4c, d). VNOs from pregnant females displayed lower numbers of BrdU^+^ cells at GD20, compared to non-pregnant controls (**P <0.01, n = 4 mice per group; Fig. 4c, d), indicating a decrease in cell survival of cells produced 20 days earlier. Therefore, the increase in neurogenesis (Fig. 4a, b, e, f) seems to be proportionally higher than the decrease of cell survival during late pregnancy (Fig. 4c, d). BrdU^+^ cell numbers at day 1 were similar in both pregnant mice and controls (*P* = 0.91; Fig. 4d), showing that proliferation is similar at this stage and mating has no major effect. Together, these results suggest that the increase of immature VSNs during pregnancy is caused by both higher proliferation and cell turn-over, but not by enhanced cell survival. This might be indicative of a cell-selective differentiation/survival process in pregnant females toward specific neuronal cell types.

We next sought to establish when pregnancy modulates neurogenesis in the maternal VNO by determining in which gestational period cell proliferation occurs. We therefore mated females with C57Bl/6 males (GD0) and collected maternal VNOs at GD1, 7, 14, and 20. We measured VNO cell proliferation following embryo implantation using immunodetection of Ki-67 (Fig. 4e, f). In these experiments, we observed a significant increase in the number of Ki-67^+^ cells only at GD20 (Fig. 4e, f). By contrast, maternal VNOs that were collected at GD1, GD7, or GD14 showed no significant increase in Ki-67^+^ cells compared to control female VNOs (Fig. 4e, f).

### The VNO transcriptomes of pregnant vs. non-pregnant females

**Fig. 5.**
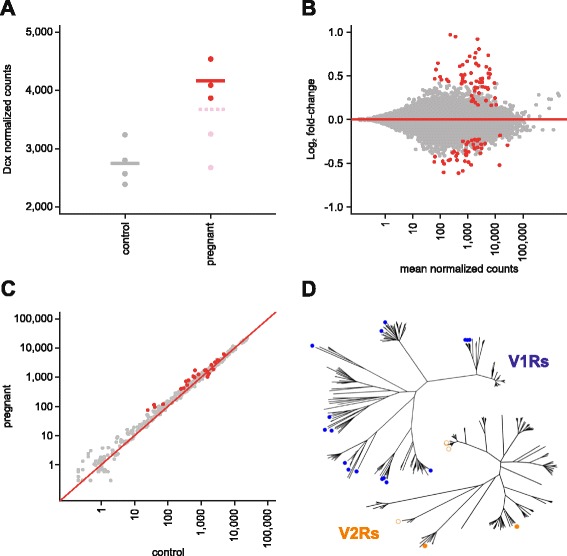
Pregnancy alters gene expression in the vomeronasal organ (VNO). (**a**) *Doublecortin* (*Dcx*) expression measured by high-throughput RNA-sequencing (RNAseq) in control and pregnant mice. The two pregnant females (pink) that did not display increased *Dcx* expression compared to controls (grey) were excluded. The three females that displayed increased neurogenesis (red) were used in further analyses. Mean expression values are indicated by horizontal lines; the dotted pink line indicates the mean when all five pregnant mice are included. (**b**) Differential gene expression of the whole VNO transcriptome of pregnant mice compared to control mice. Statistically significant differentially expressed genes (101, false discovery rate (FDR) <5 %) are highlighted in red. (**c**) A scatter plot of relative VR gene and pseudogene expression levels (normalized counts) in the pregnant versus the control mice. Statistically significant genes (24, FDR <5 %) are highlighted in red. The red line represents the 1:1 diagonal. Each differentially expressed vomeronasal receptor (VR) gene is detailed in Fig. 7. (**d**) Phylogenetic trees of all intact V1R and V2R genes, indicating the clades in which differentially expressed VR genes are located (V1Rs, blue; V2Rs, orange). Filled circles indicated VR genes upregulated in pregnant females, open circles indicate VR genes downregulated in pregnant females

To further characterize the molecular and cellular processes occurring in the VNO during pregnancy, we analyzed its transcriptome in pregnant (GD20) and non-pregnant control female mice via high-throughput RNA-sequencing (RNAseq). We sequenced five pregnant and four age-matched control VNO samples at high depth using the Illumina HiSeq platform (Additional file 2). To ensure that our RNA samples were captured from pregnant mice at the peak of VNO neurogenesis, we first analyzed the levels of *Dcx* expression (Fig. 5a). We found that three of the five pregnant females displayed ~1.5-fold increases in *Dcx* expression compared to the control mice, consistent with the observed increase in neural proliferation (Fig. 4). The other two females had no increase in *Dcx* expression. Interestingly, the embryos from these mice appeared to be 1–2 days younger than the other three litters, suggesting the development of their pregnancy was slightly delayed and the peak of neurogenesis had not yet been reached. We therefore excluded these two samples from our subsequent analyses.

**Fig. 6.**
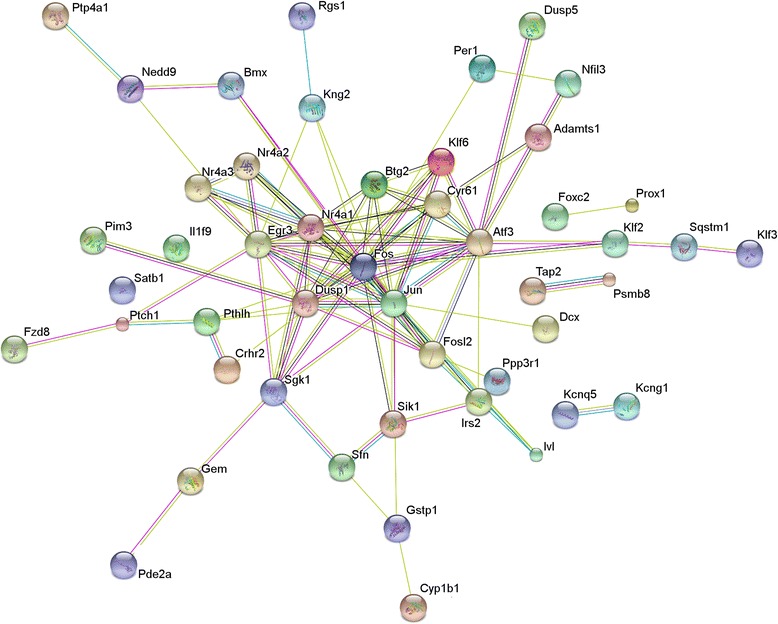
Genes that are differentially expressed in the vomeronasal organ of pregnant females are predicted to interact in a network. A STRING (http://string-db.org) analysis of 46 of the 101 differentially expressed genes that encode proteins predicted to interact with at least one other. Each protein is indicated by a circle, connecting lines indicate evidence of interaction. A red line indicates evidence of fusion; a green line is evidence of a shared neighborhood; a blue line is evidence of co-occurrence in phyletic profile; a purple line is experimental evidence of interaction; a yellow line is co-occurrence in published abstracts; a light blue line is co-occurrence in databases; a black line is evidence of co-expression

To compare the transcriptomes of the remaining three pregnant females to the controls, we first performed a differential transcriptome-wide expression analysis. In total, 101 genes were identified as significantly differentially expressed (DE) with a false discovery rate of 5 %; 59 of these were upregulated in the pregnant samples, whereas 42 were downregulated (Fig. 5b, Additional file 2). A gene ontology analysis on the DE genes revealed a significant enrichment of terms related to cell differentiation, regulation of transcription, cell proliferation, growth, and apoptosis, as well as central nervous system neuron development and axonogenesis (Additional file 2). Many of these DE genes have been reported to interact (Fig. 6) and form a regulatory network likely to underlie the observed alterations in proliferation and cell turn-over.

**Fig. 7.**
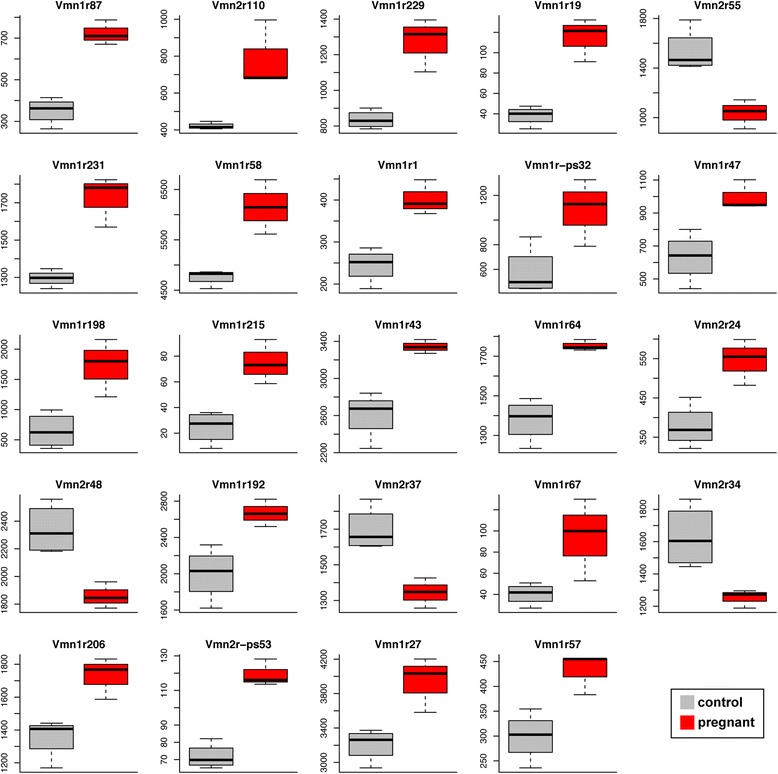
The vomeronasal receptor genes and pseudogenes differentially expressed in pregnant mice. Each of the 24 significantly differentially expressed genes from Fig. 5c (false discovery rate <5 %) are represented with a box-and-whisker plot of the RNAseq normalized counts (for both depth of sequencing and vomeronasal sensory neurons number) in control (grey) and pregnant (red) mice

We found no significant difference in the expression of the VR gene repertoire when considered together (paired *t*-test, *P* = 0.08); however, the VNO contains hundreds of different sub-types of VSNs, each defined by the expression of a given VR, or combination of VRs. If the neurogenesis or turn-over of neurons during pregnancy disproportionally affected specific VSN subtypes, the expression of the VR(s) that define those sub-types may be altered relative to the others. To test this, we normalized for the number of total mature neurons in each sample using the expression of genes specifically expressed in mature VSNs (see Methods for details). We identified 24 VR genes that were significantly DE; most (83.33 %) were proportionally upregulated in the VNOs of the pregnant mice (Fig. 5c). From these, 17 are V1R genes and 7 are V2R genes (Fig. 7), distributed across a number of VR subfamilies (Fig. 5d). Together our results indicate that pregnancy induces observable differences in gene expression indicative of alterations in cell proliferation and cell turnover in the VNO of pregnant mice. We find evidence of small biases towards a few subtypes of VSNs, but the majority of the VSN repertoire remains unchanged.

### Estrogen stimulates VNO neurogenesis

**Fig. 8.**
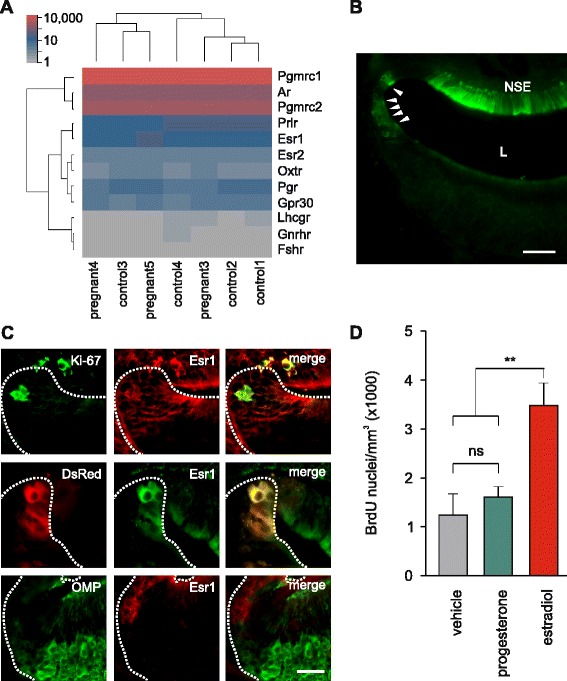
Vomeronasal organ (VNO) neurogenesis is facilitated by estrogen. (**a**) A heat map of hormone receptor gene expression in the VNO, from high-throughput RNA-sequencing (RNAseq) data. Expression values are in normalized counts. Mean *Esr1* expression is 144 normalized counts and mean *Esr2* expression is 18 normalized counts, neither are differentially expressed between pregnant and control mice. Pgrmc1, Pgrmc2, progesterone receptors membrane-component 1 and 2; Ar, androgen receptor; Prlr, prolactin receptor; Esr1, estrogen receptor α; Pgr, progesterone receptor; Esr2, estrogen receptor β; Gpr30, G-protein coupled estrogen receptor 1; Oxtr, oxytocin receptor; Lhcgr, luteinizing hormone/choriogonadotropin receptor; Gnrhr, gonadotropin releasing hormone receptor; Fshr, follicle stimulating hormone receptor. (**b**) Immunolocalization of estrogen receptor α (Esr1) in a wild type C57Bl/6 female VNO coronal section. Arrowheads indicate Esr1^+^ cells at the lateral corners of the epithelium where neuronal progenitors congregate. NSE, Non-sensory epithelium; L, Lumen. Scale bar, 50 μm. (**c**) Double labeling of VNO epithelial margins with Esr1 antibody and Ki-67 antibody (top), doublecortin (Dcx)-DsRed (central), and olfactory marker protein (OMP) antibody (bottom). VNOs from C57Bl/6 females were used for Ki-67 and OMP staining. Scale bar, 25 μm. (**d**) Quantification of BrdU^+^ cells in the VNOs of ovariectomized Dcx-DsRed females after a 9-day treatment with either, vehicle (sesame oil), progesterone, or estradiol benzoate. One way-ANOVA: F_2,24_ = 9.95; **P <0.01; control vs. progesterone *P* = 0.761; n = 8 mice (vehicle), 9 mice (progesterone), and 8 mice (estradiol). BrdU was administered 8 h before sacrifice

Neurophysiological signals that arise during pregnancy emerge in response to either embryo implantation or circulating maternal hormones. Given that the neurogenesis increase appears in the last part of gestation, we reasoned that circulating hormones might have a more central role than implantation. Using our RNAseq data, we analyzed the gene expression levels of hormone receptors in the VNO (Fig. 8a). We observed high levels of expression of the progesterone receptors membrane-component 1 and 2 (*Pgrmc1* and *Pgrmc2*), androgen receptor (*Ar*), prolactin receptor (*Prlr*), and estrogen receptor α (*Esr1*). Expression of the classical progesterone receptor (*Pgr*), as well as estrogen receptor β (*Esr2*), G-protein coupled estrogen receptor 1 (*Gper1*/*Gpr30*), oxytocin receptor (*Oxtr*), luteinizing hormone/choriogonadotropin receptor (*Lhcgr*), gonadotropin-releasing hormone receptor (*Gnrhr*), and follicle-stimulating hormone receptor (*Fshr*) was lower or not present (Fig. 8a). No significant hormone receptor expression differences were found in controls vs. pregnant females (Additional file 2).

The levels of prolactin, oxytocin, progesterone, and estrogen are known to be affected during pregnancy. Prolactin and oxytocin remain at low levels during mid-pregnancy and rise sharply near the onset of parturition and lactation [55], making them unlikely candidates for mediating the neurogenic response observed here. By contrast, circulating levels of two other hormones – progesterone and estradiol – are found at high levels during the second half of the pregnancy. Progesterone circulating levels increase following mating and drop in the last 2–3 days before parturition, and estrogen levels rise and stay high during the last half of the pregnancy [55, 56]. Pgmrc1 has been shown to be expressed in mature VSNs by immunohistochemistry [41]. Esr1, but not Esr2, protein and mRNA expression has also been reported in the VNO [57]. Our RNAseq data revealed that *Esr1* is expressed in the VNO of control and pregnant mice at similar levels and, on average, 8-fold higher than *Esr2* (Fig. 8a). We therefore analyzed the pattern of Esr1 expression in the VNO using Esr1 immunoreactivity on WT C57Bl/6 female mice. Consistent with previous studies [58], Esr1 was clearly detected in the non-sensory part of the VNO epithelium (Fig. 8b). Additionally, we detected high Esr1 immunoreactivity at the lateral corners of the VNO epithelium where neuronal progenitors congregate (Fig. 8b, arrowheads). Double-labeling experiments showed that this Esr1 immunoreactivity is co-localized with both proliferating Ki-67 cells and immature Dcx-DsRed^+^ neurons (Fig. 8c). Esr1 immunoreactivity was absent in mature OMP^+^ neurons (Fig. 8c). These results strongly suggest that estrogen could exert its effect in the VNO on neuronal progenitors by activation of Esr1.

To establish a functional link between high levels of circulating estrogen/progesterone present during late pregnancy and VNO neurogenesis, we tested the effects of estrogen and progesterone treatment on stem cell proliferation in the VNO. We performed six subcutaneous injections with estradiol benzoate (100 ng/0.1 mL/day) for a total period of 9 days into 8- to 10-week-old, ovariectomized Dcx-DsRed females. On the last day of hormone treatment we administered BrdU and extracted the VNOs after 8 h to monitor proliferation. A 9-day estradiol treatment increased BrdU-labeled cells in the VNO neuroepithelium, indicative of an increase in neurogenesis (Fig. 8d). This effect was highly specific as progesterone treatment (1 mg/0.1 mL/day) failed to induce a significant increase on the number of BrdU^+^ cells, comparable to that in vehicle-treated females (Fig. 8d). Thus, sustained high levels of circulating estrogen are sufficient to augment the proliferation of VNO neural precursors in ovariectomized females.

## Discussion

Our results demonstrate a remarkable degree of neural plasticity in sensory neurons of the VNO during pregnancy and provide new insights into the molecular basis of this phenomenon. In a comprehensive approach, we used a wide range of molecular markers and high-throughput RNA-sequencing to depict neurogenic events in the adult VNO from early progenitor cells to mature sensory neurons. We show that newly generated cells can become mature neurons in a period of 14–20 days. These cells develop a mature neuronal morphology displaying dendrites and axonal connections with central brain targets. We demonstrate that neurons generated in the adult VNO – after reaching a certain maturity stage characterized by the expression of OMP and VRs – display sensitivity to natural chemostimuli, indicating that these cells are indeed functional sensory neurons. Interestingly, the production of neural progenitors is stimulated in the VNO of female mice during the final stage of pregnancy. Employing transcriptome analysis in the VNO of pregnant vs*.* control females, we observe differential expression of 101 genes, including 24 VRs and other genes involved in regulating cell proliferation and death. Both pregnant and control female VNOs are shown to express hormone receptors, of which estrogen receptor Esr1 is prominently expressed in neural progenitor cells. Consistent with an important role of Esr1 in the neurogenic process, sustained high levels of estrogen are sufficient to increase cell proliferation in the VNO epithelium.

Estrogen receptor participation – via Esr1 receptor in particular – in cell proliferation and plasticity is well documented. Estrogen affects hematopoietic stem cell renewal during pregnancy [54], and neurogenesis in the hippocampus [26, 27, 59]. Furthermore, estrogen promotes cell survival in BNST, MeA [30], and AOB [33], and has been implicated in neuron morphological plasticity [60]. The molecular targets of estrogen and its receptors capable of influencing neurogenesis are less clear. Estrogen-induced transcriptional changes of miRNAs are candidates to mediate VNO neurogenesis. A large amount (nearly 40 % of the total) of miRNA transcription is found to be directly regulated by estrogen [61, 62], and many of these miRNAs control olfactory neurogenesis in both the main olfactory epithelium and the VNO [63].

The increase in new chemosensory neurons by pregnancy or estrogen likely has important functional consequences. The olfactory system is critical in the generation of maternal behaviors [64]. Neurogenesis in central brain areas, particularly in the hippocampus and SVZ/AOB, and its influence on parental behavior has been extensively studied (reviewed in [34]). However, the behavioral consequences of neurogenesis in nasal sensory epithelia remain largely unexplored. The VNO is specialized for recognizing social odors (pheromones and kairomones) that promote a variety of behavioral responses depending on age, gender, dominance, and hormonal status of the receiver or sender. For example, the same male major urinary protein pheromones that induce attraction in virgin females [65, 66] also provoke maternal aggression in lactating females [52], likely as a consequence of hormone-induced plastic changes in the limbic system during pregnancy, parturition, and lactation. Moreover, hormones may also modulate sensory signaling to induce behavioral changes, such as those provoked by progesterone, via directly silencing VSN responses to male major urinary proteins [41]. Similarly, enhanced neurogenesis in the vomeronasal epithelium may have an impact on maternal behaviors displayed during and after pregnancy. In fact, it has long been hypothesized that an increase in VSN turnover during pregnancy could play a key role for the duration of memory formation in the context of the Bruce effect, which is shorter in females undergoing pregnancy or when treated with estradiol implants [37]. The formation of new VSN-AOB connections during pregnancy could provide a potential mechanism to increase the likelihood of pregnancy block in females mated with a previously exposed stud male, thus diminishing female olfactory memory.

Our data indicate that relative changes in gene expression of VRs at the end of pregnancy are rather small, affecting only 6 % of the approximately 360 intact VR genes. The majority of these genes were upregulated, suggesting that this effect may be a consequence of increased neurogenesis in pregnant mice. These data are consistent with an increase in the proportion of specific sub-types of VSNs during pregnancy, perhaps those specialized in detecting maternal behavior-related pheromones. Of the VR genes upregulated here, we found that the majority belong to the V1R family (Figs. 5d and 7), and may therefore be considered as candidate receptors for mediating maternal behaviors. We cannot exclude that larger differences in receptor expression may appear at later stages, i.e. 1–2 weeks after parturition, once a larger number of newly generated neurons reach maturity. However, VNO-dependent maternal behaviors are apparent near the time of delivery, arguing that the generation of mature VSNs that detect maternal pheromones are likely to occur during late gestation. It is also possible that the proportion of VSNs remains stable during pregnancy, and the differential expression we detect is a result of transcriptional upregulation of some VR genes, but not of others. In this case, the generation of new sensory neurons during pregnancy may be related to a more fundamental physiological response, such as an increase in sensitivity or establishment of new connections with AOB targets. Similarly, changes in smell perception reported by pregnant women have been largely classified as increases in sensitivity rather than smell distortions [67]. Humans seem to lack a functional VNO – VRs and transduction molecules are pseudogenized – thus narrowing a sensory target for hormone modulation to the main olfactory epithelium. Further experiments must be conducted to determine whether a pregnancy-induced increase in neurogenesis also occurs in the main olfactory epithelium.

## Methods

### Mice

Animal care and experimental procedures were performed in accordance with the guidelines established by the animal welfare committees of the University of Saarland and Sanger Institute. Mice 6–15 weeks old were kept under standard light/dark cycle (12:12) with food and water ad libitum. Dcx-DsRed mice [C57Bl/6 J-Tg(Dcx-DsRed)14Qlu/J; JR# 009655] were generated as described [47]. Dcx-DsRed mice were crossed with mice in which the coding region and part of the 3’ non-translated region of the OMP gene was replaced by GFP as a histological reporter (OMP-GFP mice; B6.*OMP*^*tm3Mom*^/MomJ; originally JR# 006667) [48]. OMP is an abundant cytosolic protein expressed in all mature VSNs [22]. Breeding established offspring that were heterozygous for Dcx-DsRed, GFP, and OMP (Dcx-DsRed/OMP-GFP). C57Bl/6 mice served as additional controls.

### Bromodeoxyuridine (BrdU) administration

Singly-housed estrous females were mated overnight (dark phase of the light cycle) with C57Bl/6 males. All females were in proestrus at the time of mating. Injection of BrdU was done in all females at the same time. Mating was assessed by the presence of vaginal plugs. Control females were kept isolated. BrdU (Serotec) was administered the next day (GD1) to female mice of both pregnant and control groups. Two doses of BrdU (100 mg/kg diluted to a concentration of 10 mg/mL in Tris-HCl 0.05 M, pH 7.4) were injected intraperitoneally within a 4-h interval.

### Immunohistochemistry

Mice were anesthetized with ketamine (Bayer Health Care, Leverkusen) and xylazine (Pharmacia GmbH, Berlin) and perfused transcardially with 0.9 % saline solution followed by 4 % paraformaldehyde in PBS. VNOs were removed, postfixed for 2 h in 4 % paraformaldehyde, equilibrated overnight in PBS containing 30 % sucrose, embedded in OCT (Tissue-Tek), and snap-frozen in a dry ice/2-methylbutane bath. Frozen tissue sections (16 μm) were collected on glass slides (Superfrost Plus, Polysciences) and stored at −80 °C until use. All immunocytochemical procedures were conducted at room temperature, except for tissue incubation in primary antibodies (4 °C). Sections were washed three times in PBS (10 min each), incubated in blocking solution (0.5 % Triton X-100, 4 % horse serum, in PBS) for 1 h, and incubated overnight in blocking solution containing the primary antibody. The tissue was then washed thre times in PBS (10 min each) followed by incubation in secondary antibody for 1 h. Primary antibodies were: Ki-67 (1:250; mouse polyclonal, 556003, BD Pharmingen), PCNA (1:5000; mouse polyclonal, P-8825, Sigma), Dcx (1:100; goat polyclonal, sc-8066, Santa Cruz), ER-alpha (1:200; rabbit polyclonal, sc-543, Santa Cruz), OMP (1:5000; rabbit polyclonal, sc-67219, Santa Cruz), V2R2 (1:10,000; generous gift from R. Tirindelli, Univ. Parma), BrdU (1:2000; rat monoclonal, OBT00303CX, AbD Serotec). Secondary antibodies were: biotin conjugated horse anti-mouse (1:250; BA2001, Vector), goat anti-rabbit (1:250; BA-1000, Vector), and rabbit anti-rat (1:250; BA-4001, Vector); AlexaFluor 488 donkey anti-goat (1:1000; A11055, Vector) and goat anti-rabbit (1:1000; A11046, Vector). The incubation with biotin-conjugated secondary antibodies was followed by incubation with Streptavidin Alexa Fluor 488 (1:1000; S-11223, Vector). For Ki-67 and PCNA immunoreactions, antigen retrieval was performed by heating the tissue slides to 70 °C in 2 % citrate buffer (pH 7.6) for 10 min. For BrdU, tissue slides were treated with 2 N HCl at 37 °C for 1 h, followed by pH neutralization with borate buffer (pH 8.5) for 10 min. Sections were mounted using Vectashield Mounting Medium with DAPI (H-1200, Vector). Fluorescence images were acquired on an Olympus IX71 microscope. Counts of the number of cells were evaluated blindly for each animal. BrdU^+^ neurons (mean number of cells/mm^3^) were quantified in the whole VNO by averaging the counts from 8–18 non-consecutive sections per mouse.

### Confocal laser scanning microscopy

Confocal images of dendritic knobs were taken with a Leica SP5 CLSM microscope. GFP was excited at 488 nm and DsRed at 543 nm. Emission was collected between 500 and 535 nm for GFP images and between 560 and 720 nm for illustration of DsRed. The density of the dendritic knob endings at the surface of the sensory epithelium was determined manually from randomly selected areas of at least 1,000 μm^2^ in whole-mount preparations of the laterally opened VNO. Confocal images of the AOB of Dcx-DsRed/OMP-GFP mice (14 μm cryosections, sagittal plane) were taken with a Zeiss LSM 780 microscope. Images are shown as maximum intensity projections (3 μm stack), minimally adjusted in brightness and contrast using Adobe Photoshop Elements 10.

### Live-cell calcium imaging

For Ca^2+^ imaging of freshly dissociated VSNs [51, 52], VNO epithelium of adult Dcx-DsRed or Dcx-DsRed/OMP-GFP mice of both sexes was detached from the cartilage and minced in PBS at 4 °C. The tissue was incubated (20 min at 37 °C) in PBS supplemented with papain (0.22 U/mL) and DNase I (10 U/mL; Fermentas), gently extruded in DMEM (Invitrogen) supplemented with 10 % FBS, and centrifuged at 100 × *g* (5 min). Dissociated cells were plated on coverslips previously coated with concanavalin-A type V (0.5 mg/mL, overnight at 4 °C; Sigma) and loaded with the Ca^2+^ indicator fura-2/AM (5 μM; Invitrogen). Coverslips containing fura-2-loaded VSNs were placed in a laminar-flow chamber (Warner Instruments) and constantly perfused with extracellular solution. Ratiometric (F340/F380) fura-2 Ca^2+^ imaging was performed using an Olympus IX71 microscope equipped with a Hamamatsu camera. Image pairs were acquired at 0.25 Hz and analyzed using ImageJ (NIH). Chemostimuli were delivered in random order through the bath for 30 s. Post hoc immunostaining of VSNs was performed directly in the recording chamber of the Ca^2+^ imaging setup so that all responding cells could be easily identified by using the coordinates of acquired images [51, 52]. Virtually no cells were lost using this procedure. VSNs were fixed using PBS containing 4 % paraformaldehyde (10 min), incubated for 10 min in blocking solution (0.1 % Triton X-100, 1 % FBS, 50 mM NaCl, 100 mM Tris-HCl, pH 7.5), incubated with anti-V2R2 (1:10,000) or anti-Ki-67 (1:250) and then for 30 min at room temperature, and washed in blocking solution (5 min) followed by an incubation (30 min, room temperature) in secondary antibody (Alexa-Fluor 488 goat anti-rabbit, 1:500). No antigen retrieval was performed. Cell nuclei were stained (5 min, RT) with DAPI (0.5 μg/mL; Sigma).

### Chemostimulation

Chemostimuli for Ca^2+^ imaging were prepared fresh daily and diluted in Hepes-buffered Hank’s balanced salt solution (Invitrogen) giving the following final concentrations: SYFPEITHI, 5 × 10^−11^ M; HMW fraction, 1:300 dilution; urine, 1:300 dilution; 90 mM KCl. Fresh urine was collected daily from adult C57Bl/6 males (8–12-week-old, sexually naïve). Urine was size-fractionated to obtain HMW fraction from 0.5 mL of urine by centrifugation (14,000 × *g* for 30 min) using Microcon 10-kDa molecular mass cutoff ultrafiltration columns (Millipore). The centrifugation retentate was washed with 0.5 mL of PBS three times and resuspended in 0.5 mL of PBS. The major histocompatibility complex-peptide SYFPEITHI was initially dissolved in bicarbonate-containing bath solution [95 % O_2_/5 % CO_2_; 120 mM NaCl, 25 mM NaHCO_3_, 5 mM KCl, 5 mM N,N-bis[2-hydroxyethyl]-2-aminoethanesulfonic acid, 1 mM MgSO_4_, 1 mM CaCl_2_, 10 mM glucose] to give 100 μM stock solutions. Further dilutions were made in Hepes-buffered Hank’s balanced salt solution.

### Mating

Naïve Dcx-DsRed female mice were group housed (three per cage) for 2 weeks prior to mating to enhance estrous cycle synchrony. Females in proestrus were isolated and mated with an adult C57Bl/6 male for 12 h. The estrous phase was determined by vaginal smears [66]. Mating occurrence was assessed by the presence of vaginal plugs 12 h after introduction of the male (GD0). The male was then removed from the cage for the rest of the pregnancy. Females presenting plugs and that did not become pregnant (not presenting weight gain and embryos in the uterus) were used as controls. Embryo implantation might occur within 72 h after mating. We therefore estimate a maximum error of −3 days in our pregnancy time line. For male bedding exposure, 150 mL of soiled C57Bl/6 adult male bedding was added every second day in the home cage of naïve, singly housed Dcx-DsRed adult females.

### RNAseq data processing and analysis

VNOs from pregnant and control females were dissected and immediately frozen on dry ice. RNA was extracted using the RNeasy mini kit (Qiagen) with on-column DNAse digestion. mRNA was prepared for sequencing using the TruSeq RNA sample preparation kit (Illumina). All samples were multiplexed together and sequenced on four lanes on the Illumina HiSeq 2500 platform, to generate 100 bp paired-end reads. All data is available through the European Nucleotide Archive and the corresponding accession numbers can be found in Additional file 2. Sequencing reads were mapped using STAR 2.3 to the GRCm38 mouse reference genome, annotation version 72 in the *Ensembl* mouse genome database. The annotation file was modified to substitute the gene models for the olfactory and vomeronasal receptors by those described previously [68], which include extended UTRs and additional non-coding exons. The number of fragments aligned to each gene was counted using the HTSeq package, with the script *htseq-count* (mode *intersection-nonempty*). Any reads that map to multiple locations in the genome (also called multireads) were not counted towards the expression estimates since they cannot be assigned to any gene unambiguously. To compare the expression values across samples, raw count data was normalized to account for the depth of sequencing. Size factors were calculated using DESeq2’s function *estimateSizeFactorsForMatrix*, and raw counts were divided by the corresponding size factor for each sample. In order to analyze the molecular influence of neurogenesis on VR genes we used expression of Dcx as criterion to determine the precise neurogenesis peak. Pregnant females displaying less than 1.5-fold increases in Dcx expression compared to the control mice were not considered to be at the peak of neurogenesis and were therefore excluded from further analyses. To test for differential expression, DESeq2 was used with standard parameters. Genes were considered to be differentially expressed if they had an adjusted *P* value of ≤0.05 (equivalent to a false discovery rate of 5 %). To assess the relative proportion each VR gene contributes to the total VR repertoire, the geometric mean of three VSN-specific genes (*Trpc2*, *Omp*, and *Tubb3*) was calculated. These genes display consistent ratios of expression across all samples, and were therefore used to calculate size factors to normalize the VR expression estimates (which had previously been normalized for depth of sequencing). All VR genes were then tested for differential expression with DESeq2, providing the algorithm with size factors of 1 for all samples, to avoid further normalization. To find terms that are enriched in our list of DE genes the over-/under-representation algorithm from GeneTrail (http://genetrail.bioinf.uni-sb.de/) was used. The background provided were all those genes tested for differential expression. To assess whether the DE genes form putative regulatory networks, STRING (http://string-db.org/) was used with default settings, for the 101 DE genes only. All normalized data and detailed results of the DE and enrichment analyses can be found in the Additional file 2.

### Ovariectomy

Individually caged 6–8 week old mice were anesthetized with isoflurane (Abbott). Following a skin dorsal midline incision, caudal to the posterior border of the ribs, muscles of the posterior abdominal wall were separated to enter the abdominal cavity. Blunt subcutaneous dissection, lateral to the skin incision, was made beneath the muscle layer to gain access to the ovaries and remove them. The uterine horn was reintroduced into the abdomen and the process was repeated bilaterally. The muscle incision was sutured and the skin incision closed using wound clips.

### Hormonal treatments

Two weeks after ovariectomy, mice were injected with estradiol benzoate or progesterone as previously described [31]. Briefly, either estradiol benzoate (100 ng/100 μL; Sigma) or progesterone (1 mg/100 μL; Sigma) were dissolved in sesame oil (Sigma) and injected subcutaneously at days 1–2 and 6–9. At the end of the hormonal treatment (day 10), two doses of BrdU (100 mg/kg diluted to a concentration of 10 mg/mL in Tris-HCl 0.05 M, pH 7.4) were administered intraperitoneally with a 4-h interval between the two administrations.

### Statistics

Independent Student’s *t*-test was used for measuring the significance of difference between two independent distributions. Multiple groups were compared using either one-way or two-way analysis of variance (ANOVA) with Tukey’s test as a post hoc comparison. Analysis was performed using Origin8.6 (OriginLab) software. Unless otherwise stated, results are presented as means ± SEM.

## Conclusions

In summary, our study contributes to determine the developmental profile of VSNs and the modulation of neurogenesis by pregnancy, and directly demonstrates an effect of estradiol on this neurogenesis, which may serve to explain pregnancy-evoked changes in odor-guided behaviors. These results reinforce the view that factors including hormonal state and sensory input have the potential to finely tune sensory systems to adapt them to changing biological contexts.

## Additional files

Additional file 1:
**Excel workbook containing the raw data from Fig.** **4b**, **d** **and** **f**. (XLSX 12 kb) Additional file 2:
**Excel workbook containing the mapping statistics of the RNAseq data for each sample, along with the accession numbers for the raw data; the normalized counts for the whole transcriptome; the differential expression analysis between the pregnant and control samples; the gene ontology categories significantly overrepresented in the differentially expressed genes; the normalized counts for the VR genes, accounting for total VSN number; and the differential expression analysis on the VR genes only, after normalization for VSN number, between the pregnant and control samples.** The column ‘length’ corresponds to the total exonic bases in all transcripts from the gene. For the differential expression sheets, the columns contain the following data: ‘baseMean’, corresponds to the mean normalized expression value for the gene across all samples; ‘log2FoldChange’, is the fold change between the pregnant and control samples, log_2_ transformed; ‘lfcSE’, corresponds to the standard error associated with the fold change estimation; ‘stat’, is the Wald statistic; ‘pvalue’ is the *P* value of the test; and ‘padj’ is the *P* value after adjusting for multiple testing (Benjamini-Hochberg). Genes that have both their ‘pvalue’ and ‘padj’ set to NA contain outliers; genes with only their ‘padj’ set to NA were filtered prior to the test because their normalized counts were too low. (XLSX 11150 kb)

## Abbreviations

AOB: Accessory olfactory bulb
BrdU: Bromodeoxyuridine, 5-bromo-2'-deoxyuridine
Dcx: Doublecortin
DE: Differentially expressed
Esr1: Estrogen receptor α
GD: Gestation day
GFP: Green fluorescent protein
HMW: High molecular weight urine fraction
miRNA: MicroRNA
OMP: Olfactory marker protein
PCNA: Proliferating cell nuclear antigen
RNAseq: High-throughput RNA-sequencing
SVZ: Subventricular zone
VNO: Vomeronasal organ
VR: Vomeronasal receptor
VSNs: Vomeronasal sensory neurons
